# (+)-Dehydroabietic Acid, an Abietane-Type Diterpene, Inhibits *Staphylococcus aureus* Biofilms *in Vitro*

**DOI:** 10.3390/ijms140612054

**Published:** 2013-06-05

**Authors:** Adyary Fallarero, Malena Skogman, Janni Kujala, Mohanathas Rajaratnam, Vânia M. Moreira, Jari Yli-Kauhaluoma, Pia Vuorela

**Affiliations:** 1 Pharmaceutical Sciences, Department of Biosciences, Abo Akademi University, Artillerigatan 6 A, 3rd floor, Biocity, FI-20520, Turku, Finland; E-Mails: adyary.fallarero@abo.fi (A.F.); malena.skogman@abo.fi (M.S.); jkujala@abo.fi (J.K.); 2 Division of Pharmaceutical Chemistry, Faculty of Pharmacy, University of Helsinki, Viikinkaari 5 E, P.O. Box 56, FI-00014, Helsinki, Finland; E-Mails: smohanathas@inbox.com (M.R.); vania.moreira@helsinki.fi (V.M.M.); jari.yli-kauhaluoma@helsinki.fi (J.Y.-K.); 3 Division of Pharmaceutical Biology, Faculty of Pharmacy, University of Helsinki, Viikinkaari 5 E, P.O. Box 56, FI-00014, Helsinki, Finland

**Keywords:** anti-biofilm, inhibitor, biofilm, *Staphylococcus aureus*, abietic acid, natural compounds, phenotypic assays, (+)-dehydroabietic acid, screening

## Abstract

Potent drugs are desperately needed to counteract bacterial biofilm infections, especially those caused by gram-positive organisms, such as *Staphylococcus aureus*. Moreover, anti-biofilm compounds/agents that can be used as chemical tools are also needed for basic *in vitro* or *in vivo* studies aimed at exploring biofilms behavior and functionability. In this contribution, a collection of naturally-occurring abietane-type diterpenes and their derivatives was tested against *S. aureus* biofilms using a platform consisting of two phenotypic assays that have been previously published by our group. Three active compounds were identified: nordehydroabietylamine (**1**), (+)-dehydroabietic acid (**2**) and (+)-dehydroabietylamine (**3**) that prevented biofilm formation in the low micromolar range, and unlike typical antibiotics, only 2 to 4-fold higher concentrations were needed to significantly reduce viability and biomass of existing biofilms. Compound **2**, (+)-dehydroabietic acid, was the most selective towards biofilm bacteria, achieving high killing efficacy (based on log Reduction values) and it was best tolerated by three different mammalian cell lines. Since (+)-dehydroabietic acid is an easily available compound, it holds great potential to be used as a molecular probe in biofilms-related studies as well as to serve as inspirational chemical model for the development of potent drug candidates.

## 1. Introduction

Bacterial biofilms is the term used to describe the surface-attached bacterial lifestyle. Cells in biofilms grow as communities, surrounded by a self-produced thick layer of extracellular polymeric substances (EPS, also known as matrix or slime). Thus, biofilms are structurally different from single-cells (planktonic) bacteria. These differences in the physical architecture between biofilms and single-cell bacteria also relate to their differences in functional behavior. The presence of the matrix, for instance, protects cells in biofilms from the detrimental effects of chemical insults. In fact, due to the presence of the matrix as well as slower growth rate and resistant subpopulations, biofilms possess much lower susceptibility to antimicrobial therapy or biocides [1–3].

Biofilms are involved in a wide range of infections such as periodontitis, otitis, cystic fibrosis, urinary tract infections, osteomyelitis and medical device-associated infections, and they account for an overwhelming proportion of the persistent, antibiotic-resistant infections [4,5]. Because the resistance mechanisms in biofilms are of multifactorial nature, drug discovery efforts need to be guided in various directions. Methodology-wise, the search for anti-biofilm compounds has been embraced by using target-directed, phenotypic-based as well as *in silico* approaches (for a recent review see [6]). Using small molecular weight compounds that can easily penetrate the biofilms without significant chemical degradation is still a largely pursued strategy, on the rationale that such compounds can decrease the burden of viable cells, and/or sensitize biofilms to conventional anti-biofilm therapy. However, so far, still a limited repertoire of easily available compounds has been reported that can selectively act *in vitro* and eradicate existing biofilms at low concentrations, especially in the case of those formed by *Staphyloccocus aureus* [7]. Moreover, only one disinfectant (commercially sold by Sterilex, a US-based company) and no antibiotic has been approved by a regulatory agency, to use specifically against bacterial biofilms. Lacking of potent controls is also a common scenario in basic biofilm studies, since even millimolar concentrations of antibiotics are not enough to cause high inhibitory effects [1,8]. Thus, from a basic research perspective, the limited repertoire of active compounds that can be used as chemical tools or probes limits target validation studies, as well as *in vitro* or *in vivo* studies aimed at exploring biofilms behavior and functionality. From a drug discovery viewpoint, the lacking of drug candidates places us in a very vulnerable position to face infectious diseases. Thus, expanding antibacterial discovery to the search for new anti-biofilm compounds seems mandatory for current pharmaceutical and biomedical research.

One of the major defenses of coniferous plants is the secretion of oleoresin, which is a complex mixture of terpenoids consisting of turpentine (monoterpene and sesquiterpene) and rosin (diterpene) fractions. If the plant is injured, the turpentine acts as the solvent that transports compounds from the rosin fraction to the damaged site. Once exposed to atmospheric conditions, the volatile turpentine evaporates leaving the diterpenoid resin acids. The resin acids polymerize to form a chemical barrier that seals the wound while trapping insect invaders and microbial pathogens [9]. A complex mixture of diterpenoid resin acids is obtained in bulk quantities from industrial manufacture of cellulose using the Kraft process, and various resin acid mixtures are commercially available. In addition, the resin acids can be obtained directly from the coniferous rosin. Some representatives of this class of abietane-type diterpenoids are abietic acid, neoabietic acid, palustric acid, pimaric acid and isopimaric acid.

Antibacterial effects of the diterpenoid resin acids have been indeed studied, specifically against methicillin-resistant *S. aureus* strains [10,11] and in particular of abietane-type diterpenes [12]. However, the investigation on the anti-biofilm properties of these compounds has been very scarce, and only two studies have addressed it, resulting in the identification of two anti-biofilm compounds: 4-*epi*-pimaric acid [13] and salvipisone [14]. Thus, although still largely unexplored, it seems likely that diterpenoid resin acids compounds can display anti-biofilm properties.

Here, we attempted to find and characterize compounds acting on *S. aureus* biofilms from a collection of abietane-type diterpenes containing relatively simple resin acids that have not been previously studied. These compounds are easily available, chemically stable and cost-effective which altogether benefit their wider exploitation as potent anti-biofilm probes in laboratory trials. Furthermore, focusing on these compounds allows the revalorization of a previously neglected product from the forest industry (wood rosin), which is a desirable strategy towards the discovery of more environmentally friendly biocides.

## 2. Results and Discussion

*S. aureus* is a very versatile pathogen due to its various resistance mechanisms and it is frequently associated to hospital-acquired infections, which are often related to biofilms in medical devices [15–17]. Promising anti-biofilm compounds have been described, but a recurrent feature of these studies is that they typically focus only on preventing staphylococcal attachment. Several compounds have been reported to prevent bacterial colonization on different surfaces without killing planktonic cells [18–20]. Among them, furanones, a naturally occurring class of compounds, have shown ability to prevent bacterial adhesion, which could be related to some extent to their inhibitory effects of quorum sensing in *Staphylococcus* spp. [21]. These compounds could be useful for coating medical devices. However, a great need also exists for easily available compounds able to act on existing biofilms to counteract the infection once it has been started.

### 2.1. Identification of Three Anti-Biofilms Compounds

In this contribution, 10 abietane-type diterpenes, including the commercially available (+)-dehydroabietic acid and (+)-dehydroabietylamine as well as eight other semi-synthetic derivatives (Table 1 and the Appendix), were screened for anti-biofilm activity against *S. aureus* biofilms using a screening platform developed by us [8].

Compounds were tested by adding them before biofilms were formed (prior-exposure) or after forming them (post-exposure) and effects on viability and biofilm biomass were simultaneously measured in both exposure types, using resazurin and crystal violet staining assays, respectively. Given the very low rate of positive hits that has been previously detected in our laboratory against this strain [22] and in order to increase the chances of identifying hits (even weak ones), we chose to perform this initial screening at an unusually high concentration of 400 μM, when compared to the concentrations that are used for primary screenings, typically within the low micromolar range (1–10 μM). Nordehydroabietylamine (**1**), (+)-dehydroabietic acid (**2**), and (+)-dehydroabietylamine (**3**) were shown to significantly prevent biofilm formation and to act on formed *S. aureus* biofilms with potencies confirmed in the micromolar range (Table 2), whereas none of the urea derivatives (**4**–**10**) were active.

Potency values obtained for the three active compounds **1**–**3** were consistent when measuring their effects in biofilm biomass and viability on the two exposure paradigms (Table 2). Anti-biofilm potency assessments revealed that positive effects on existing biofilms were displayed at only 2 to 4 times greater concentrations of the compounds, if compared to the ones needed to prevent biofilms establishment. This is an important advantage as opposed to typical antibiotics, for which up to 1000-fold higher concentrations are needed for eradication of mature biofilms [1,3,23,24]. The order of compound activity in both exposure paradigms was: **1** < **2** < **3**.

### 2.2. Antibacterial versus Anti-Biofilm Effects of Compounds **1**–**3**

To determine whether the detected effects in prevention of biofilm formation could be at least partially related to antibacterial effects on suspended cells, MIC values were calculated (Table 2). Although the three compounds were effective against planktonic bacteria, their MIC values were higher than those concentrations causing 50% inhibition of biofilm formation, thus indicating that preventive effects on biofilm formation can be caused by non-bacteriostatic mechanisms. The best selectivity towards *S. aureus* biofilms was displayed by compound **2**, which had a MIC value of 70 μM but prevented 50% of biofilm formation (IC_50_) at 30 μM and reached 90% of biofilms inhibition (IC_90_) at a sub-MIC value of 60 μM. In contrast, 4-*epi*-pimaric acid was reported to prevent biofilm formation by the oral pathogen *Streptococcus mutans*, at a concentration of 13.2 μM, which was only half its MIC value determined on suspended cells [13]. On the other hand, salvipisone was shown to prevent the adhesion of *S. aureus* (ATCC 29213) at a concentration of 60 μM, which coincided with its bacteriostatic effects, measured by MIC values [14]. Thus, selectivity of compound **2** is better than that of the only two anti-biofilm abietane-type diterpenes reported in the literature so far.

### 2.3. Non-Specific Cytotoxicity on Mammalian Cells

Prior research on resin acids had raised concerns on the possible occurrence of cytotoxicity in mammalian cells. For instance, abietic acid had been shown to be toxic to pulmonary epithelial cells, causing cell lysis [25]. To exclude the possibility that **1**–**3** could be exerting anti-biofilm effects due to non-specific cytotoxicity (*i.e.*, due to a membranolytic effect), cytotoxicity assays were performed. These cell lines (HL, HepG2 and GT1-7) were selected for these studies as they represent three distinct cellular phenotypes, originating from human respiratory tract, human hepatocytes and mouse hypothalamus, respectively. Together, they offer a good predictive assessment of possible cytotoxic effects that may occur upon exposure to compounds **1**–**3**. After 24 h, concentrations of **1** and **3** above 25 and 5 μM respectively were shown to cause more than 50% cell death. In contrast, the three cell lines tolerated **2** better, and estimated LC_50_ values were (between parenthesis, 95% confidence intervals): 106.4 μM (99.6–113.7 μM) for HL cells; 153.0 μM (139.9–167.3 μM) for HepG2 cells, and 176.3 μM (165.4–188.0) for GT1-7 neurons (Table 3). Compound **2**, (+)-dehydroabietic acid, has been reported to exert a potent gastroprotective effect *in vivo*, and LC_50_ values in human lung fibroblasts (MRC-5) and a human epithelial gastric cell line (AGS) have been estimated to be of 171 and 95 μM, respectively [26], in agreement with the results reported here. Compound **2** was then selected for follow-up studies.

### 2.4. Anti-Biofilm Killing Efficacy of Compound **2** and Biocompatibility Index

**Figure 1:**
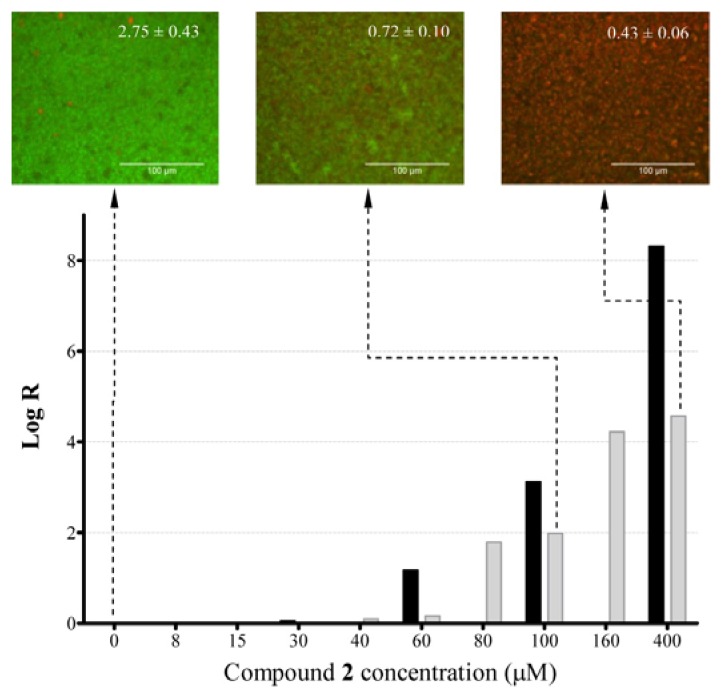
Killing anti-biofilm efficacy of compound **2** measured using Log Reduction (Log R), prior to biofilm formation (black bars) or post-biofilm formation (grey bars). As an insert, fluorescence microscopy images of 18 h *S. aureus* biofilms treated (or not) with **2** are shown. Fluorescence signal (G/R fluorescence ratios) calculated on replicate wells, are shown in each image.

The killing efficacy of compound **2** was studied by assessing the actual concentration of biofilms left on the wells upon treatment with the compound and calculating the log R parameter, as in [27] (Figure 1).

Viable bacterial concentration recovered from the untreated biofilm wells was 10^8^ CFU/mL (1.25 × 10^7^ CFU/cm^2^) and their viability was illustrated by the prevalence of green fluorescence emission in FM imaging (insert, Figure 1). As expected, for the preventive effect, compound **2** at 60 μM (corresponding to IC_90_) caused 1-log biofilm reduction, but a moderate concentration of 100 μM resulted in a 3-log reduction and a full 8-log reduction was measured at 400 μM, indicative of the highest possible killing efficacy that resulted in a complete absence of viable colonies in the treated-biofilms. Compound **2** decreased also bacterial viability on existing biofilms in a concentration-dependent manner. At moderate concentrations (80; 100 μM), a 2-log reduction was detected. A concentration of 140 μM caused a 3-log reduction, indicative of 99.9% killing of the viable cells residing on the biofilms, and higher concentrations (200; 400 μM) caused a significant 4-log reduction in viable counts. In contrast, penicillin only caused 1-log reduction at 400 μM in 18 h biofilms. In immunotolerant biofilm infections, a reduction of the biofilm burden of 3-log is highly desirable to assist the immune system in clearing the remaining pathogens *in vivo*. In parallel, upon biofilms exposure to **2** at 100 and 400 μM, a progressive shift towards red fluorescence (indicative of uptake of propidium iodide by dying cells) was registered with a decrease in the G/R fluorescence ratio to 0.72 ± 0.10 and 0.43 ± 0.06 from 2.75 ± 0.43 in untreated biofilms (insert, Figure 1). This dramatic decrease of the G/R ratio caused by the addition of higher concentrations of compound **2** is in agreement with its good killing capacity (log R results). In contrast, in biofilms that were treated with penicillin at 400 μM, the G/R fluorescence ratio decreased only to 1.54 ± 0.17, confirming that this concentration causes roughly only a 50% reduction of biofilms viability on 18 h biofilms.

To quantitatively assess the relevance of the anti-biofilm killing efficacy of compound **2** in comparison to the cytotoxicity results, the Biocompatibility Index (BI) was calculated (Table 3).

BI, as originally defined in [28], is a dimensionless parameter resulting from the ratio of the *in vitro* cytotoxicity values (in this case, half-lethal concentrations, LC_50_) to the concentration of the compound causing a 3-log reduction in the viable counts of suspended bacteria. Here, we applied this calculation to assess the impact of the anti-biofilm activity of compound **2**. The original definition presented in [28] was proposed for antiseptic agents that are expected to act within a very short exposure time. Thus, the authors used a 30-minute exposure time to the biocides for both cytotoxicity and antibacterial assays. However, in our assays, a 24-h exposure time has been used in both cases, which is the typical *in vitro* exposure time used for antibacterial assessment of antibiotics. Additionally, because the concentrations at which anti-biofilm compounds can act on existing biofilms are of more clinical relevance instead of those preventing their formation, we selected the concentration of compound **2** causing a 3-log reduction in the post-exposure paradigm (140 μM) according to the results of the log R assay (Figure 1). Although the choice of the concentration of **2** at which a 3-log reduction is achieved during the pre-exposure assay (100 μM) would have resulted in a better BI value, the authors felt that it would have not been an accurate reflection of the most challenging scenario that should be tackled *in vivo*.

The BI value of compound **2** was higher than 1 when 2 out of the 3 cell lines were used for the calculations, which is indicative of an adequate combination of an effective anti-biofilm activity with a low cytotoxicity. In the case of the HL cells, the lower LC_50_ value registered caused the BI to be lower than 1. For compound **2** to be developed into a drug, the BI values should obviously be favorable in all cases to minimize the risks of toxic effect on the host organisms. However, the usefulness of this compound as a chemical tool in basic biofilms studies is not by any means compromised with these results. Calculations of BI index are not commonly seen within reports of anti-biofilm molecules, but this represents a useful parameter for quick exclusion of undesired toxic scaffolds or weak hits in early stage of development of anti-biofilm molecules.

### 2.5. Mechanistic Insights into Anti-Biofilm Activity of Compound **2**

The question then arose as to which stage during the biofilm formation process, compound **2** would be more prone to act, and to elucidate this we performed mechanistic studies. The first experiment was designed to study the possible effects of compound **2** on the bacterial attachment to the polystyrene surface of microtiter well plates, as this is the first recognized step leading to biofilm establishment [29]. Bacteria were let to attach for 1 h in the presence or absence of compound **2**, and after 1 h, the planktonic cells were removed and replaced with fresh culture media for additional 17 h. The amount of cells that attached during the first hour was enough to ensure biofilm formation in fresh media after 17 h, and similar growth was in fact observed when compared to normal experimental conditions during 18 h without media refreshment (results not shown). For this test, compound **2** was tested at 30 and 60 μM (both concentration values are sub-MIC). Exposure to compound **2** during the attachment phase resulted in 97.5% ± 3.4% (at 30 μM) and 91.3% ± 18.7% (at 60 μM) of viable biofilms, thus no anti-biofilm activity was detected. This suggests that compound **2** does not likely target the initial bacterial attachment phase.

**Figure 2:**
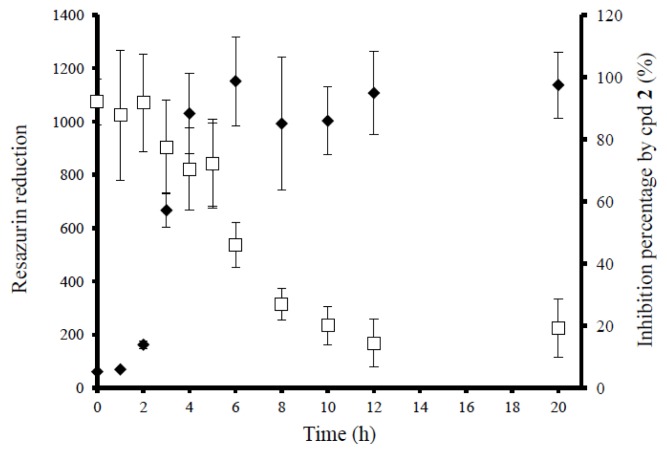
Inhibitory effects registered by the addition of **2** (unfilled squares) at different time-points, corresponding to different phases of biofilm growth by ATCC 25923 in 96-well plates (filled diamonds).

Next, the effects of **2** were studied on the proliferation and maturation phases (Figure 2). After initial attachment, bacterial cells start to divide and transition into biofilms, and this is also often referred to as the proliferation or accumulation phase. If conditions are suitable, maturation takes place, in which a plateau phase is achieved with decreased metabolic activity. The kinetics of biofilm formation (Figure 2, filled diamonds) showed that after the attachment phase (first hour), an active accumulation phase followed (up to five hours), with a plateau phase that was reached after six hours. Due to the presence of biofilms on different growth phases, a higher concentration of **2** (60 μM) was added in this trial (Figure 2, unfilled squares). At time zero, the concomitant addition of **2** and bacteria caused 90% of biofilm inhibition, corresponding to preventive effect. After that, the anti-biofilm activity of **2** was maintained at high levels (around 70% inhibition) even if **2** was added up to five hours after biofilm formation had started, during the accumulation phase. This stage is characterized by cell-to-cell adhesion, proliferation (increase in cellular density of the biofilm) and EPS production, which could all be targeted by this molecule. However, if present only during the plateau, its anti-biofilm activity decreased.

Since these effects of **2** were detected at a sub-MIC concentration (60 μM), it seems likely that this molecule targets events that are specific to the biofilm accumulation stage, such as the quorum-sensing (QS) process. However, because this compound can affect bacterial growth at higher concentrations, it is probable that it also modulates *off*-target (secondary) processes not specific to the biofilm lifestyle. For instance, 14-isopropylpodocarpa-8,11,13-trien-13-ol (totarol) which has a tricyclic skeleton similar to **2**, has been shown to display antibacterial effects via the inhibition of the oxidation of NADH and NADH related reductases [30]. In the case of **2**, the prevailing effect (anti-biofilm or antibacterial) is likely to be influenced by the concentrations reaching the biofilms or the affinities on the actual target molecules, among others. A combination of anti-biofilm and antibacterial actions has been similarly described for 4-*epi*-pimaric acid [13], which was shown to inhibit active *S. mutans* biofilm accumulation phase without affecting the adherence of the cells to polystyrene plates. This compound was shown to inhibit the synthesis of glucan (from the *Streptococcus mutans* matrix) but it also permeabilizes membranes, leading to biofilm killing and, at higher concentrations, to antibacterial effects [13].

Across all the studies performed in this study, biofilm formation was measured in the bare polystyrene surface of the microtiter well plates. However, in practice, biofilms are frequently formed on substrates (*i.e.*, catheters or other medical devices) that are conditioned with adhesive matrix proteins, such as fibrinogen, fibronectin or collagen. Consequently, testing the activity on biofilms formed on preconditioned surfaces is particularly relevant, from a clinical perspective. To mimic such scenario *in vitro*, biofilms were formed on polystyrene plates that were coated with fibrinogen and fibronectin, which are two typically abundant proteins in host cells and to which *S. aureus* contains several receptors [31–33]. Biofilm formation was quantitatively similar in protein-coated and uncoated polystyrene plates, within the whole concentration range tested (results not shown). The same inhibitory effect (around 50%) was registered for **2** (at 30 μM, corresponding to IC_50_ in the pre-exposure assay) in protein-coated or uncoated plates. For instance, in the presence of fibrinogen or fibronectin (5 μg/mL), viability in **2**-treated biofilms was 57.8% ± 7.5% and 52.3% ± 1.2%, respectively.

## 3. Experimental Section

### 3.1. Compounds Collection—Chemistry

A collection of ten diterpenes from the genus *Picea* and their derivatives was studied (Table 1), including the commercially available (+)-dehydroabietic acid and (+)-dehydroabietylamine as well as eight other semi-synthetic derivatives. Detailed information is included in the Appendix.

### 3.2. Biofilm Formation Assay

Model biofilm forming bacterium was *S. aureus* (ATCC 25923). Biofilm formation assay was conducted as previously described by our group [34]. Briefly, exponentially growing bacteria were added to flat-bottomed 96-well microplates (Nunclon Δ surface, Roskilde, Denmark) at the initial concentration of 10^6^ CFU/mL (200 μL) and biofilms were formed at 37 °C, 200 rpm for 18 h in tryptic soy broth (TSB, Fluka Biochemika, Buchs, Switzerland).

### 3.3. Compounds Exposure

Anti-biofilm effects of the compounds (in 2% DMSO) were examined prior to biofilm and post-biofilm formation. In the first case, compounds and bacterial suspension were added simultaneously and effects were examined after incubation at 37 °C, 200 rpm for 18 h. In the second case, biofilms were formed during 18 h (37 °C, 200 rpm), compounds were added and plates were incubated for 24 h at 37 °C, 200 rpm. Untreated biofilms (only exposed to culture media), cell-free wells containing only TSB and wells containing biofilms and 2% of DMSO were included as controls. Penicillin G, used as control antibiotic in all experiments, was prepared in Müeller-Hinton Broth (MHB). Initial screening of the chemical collection was conducted at a final compound concentration of 400 μM. Anti-biofilm potencies of **1**–**3** were measured within a concentration range of 1 nM–400 μM. Penicillin was tested at 400 μM unless indicated otherwise.

### 3.4. Quantification of Biofilms

Biofilms viability was measured with a redox dye, resazurin (Sigma-Aldrich, St. Louis, MO, USA) [22]. Planktonic suspension was removed, resazurin 20 μM was added and plates were incubated (200 rpm, 20 min, RT, darkness) followed by fluorescence measurement (λ_ex_ = 570 nm; λ_em_ = 590 nm) using Varioskan reader (Thermo Fisher Scientific, Vantaa, Finland). Total bacterial biomass was assessed with crystal violet dye (Sigma-Aldrich, St. Louis, MO, USA). Since resazurin is not toxic to the cells, the crystal violet assay was conducted right after finishing the viability assay as optimized in [8]. The biomass assay was performed in automated conditions [34], combining a Biomek 3000^®^ liquid handling workstation (Beckman Coulter Inc., Fullerton, CA, USA) with a Multidrop^®^ Combi dispenser (Thermo Fisher Scientific, Vantaa, Finland). The absorbance of crystal violet-stained biofilms (λ = 595 nm) was measured using Varioskan plate reader.

### 3.5. Bacteriostatic Effect on Planktonic Cells

Exponentially grown *S. aureus* suspensions (10^6^ CFU/mL) were added to the plates and incubated with serial dilutions of compounds **1**–**3** at 37 °C for 18 h, coinciding with the time used in the biofilm formation studies. After incubations, the minimal concentrations of the compounds causing no turbidity were recorded as the MIC values. MIC values were also recorded for the control antibiotic (penicillin G). Quantitative absorbance measurements (λ = 620 nm) were done using Varioskan plate reader.

### 3.6. Cytotoxicity Assessment on Mammalian Cells

Human lung (HL), HepG2 (ATCC HB-8065) and GT1-7 neurons were grown as in [35]. Cell suspensions (4 × 10^5^ cells/mL) were added into plates and incubated at 37 °C for 24 h. Then, 20 μL of culture media was replaced with a similar volume of solutions containing the compounds at different concentrations (1 nM–400 μM) and plates were incubated for another 24 h at 37 °C. Then, culture media was replaced with a solution of resazurin (20 μM) for HL and HepG2 cells and 25 μM for GT1-7 cells, and they were incubated for 2 h at 37 °C. Reduced resazurin fluorescence was measured as described in 4.4. Untreated cells, cell-free wells, as well as cells exposed to 0.5% DMSO (solvent control), were all included.

### 3.7. Studies on the Anti-Biofilm Activity of Compound **2**

#### 3.7.1. Determination of Log Reduction (Log R)

Log R was estimated as in [27], using viable cell counts in tryptic soy agar (TSA) plates. At the end of the exposure to **2**, suspensions were removed and biofilms were scraped off the wells, twice. The resulting suspension was mixed vigorously and sonicated for 5 min in order to disperse bacterial aggregates. To confirm that all biofilms have been recovered, the scrapped wells were stained with resazurin (as in 4.4). Serial dilutions were spread onto TSA plates and incubated at 37 °C overnight. Log R was determined by subtracting the log of the bacterial concentrations in treated samples from the ones quantified in untreated biofilms.

#### 3.7.2. Imaging with Wide-Field Fluorescence Microscopy (FM)

Viability kit (LIVE/DEAD^®^*Bac*Light™, Eugene, OR, USA), containing SYTO 9 and propidium iodide was used. Biofilms were formed as in 4.2, followed by the addition of **2** (100; 400 μM) for 24 h. Planktonic cells were then removed. The probes mixture (SYTO 9 5 μM; propidium iodide 30 μM) was added (6 μL) followed by incubation (15 min, RT, darkness). FM was performed with an Axiovert 200 M inverted microscope (Carl Zeiss MicroImaging, Jena, Germany) with a 40×/0.6 LD-Plan Neofluar objective and using FITC and TRITC filter sets for SYTO 9 and propidium iodide, respectively, as in [8]. Images were acquired by using a Hamamatsu ORCA-ER camera (Hamamatsu Photonics K.K., Hamamatsu, Japan) with Axiovision Release 4.8 software (Carl Zeiss Microimaging, Jena, Germany). These experiments were performed at the Cell Imaging Core, Turku Centre for Biotechnology, Finland. The fluorescent signal generated by the mixture of probes in this experiment was quantified in parallel by adding 200 μL of the probes mixture (SYTO 9 5 μM and propidium iodide 30 μM) to 96-well plates. The measurement was conducted (λ_ex_ = 485 nm and λ_em_ = 530 nm for SYTO 9 and λ_em_ = 630 nm for propidium iodide) using a Varioskan multimode plate reader.

#### 3.7.3. Effect on Initial Bacterial Adhesion

*S. aureus* suspensions (10^6^ CFU/mL) were added to the plates in the presence or in the absence of **2** (30 μM and 60 μM). Cells were let to attach for 1 h prior to removing the planktonic phase and replacing it with fresh TSB. After that, biofilms were let to form for additional 17 h before quantification with resazurin, as in 4.4.

#### 3.7.4. Effects on Biofilm Proliferation or Maturation Phases

To first confirm the time-course of biofilm formation, untreated bacterial samples (10^6^ CFU/mL) were added to the plates, and viability was measured at various time points up to 18 h. Then, in another set of samples, compound **2** (60 μM) was added at various time points after adding the suspended bacteria into the plates. The biofilm viability was quantified with resazurin at the end of the 18 h (as described in 4.4), and compared to samples that were only exposed to culture media at similar time-points.

#### 3.7.5. Bacterial Adhesion in the Presence of Host Proteins

Plates were pre-treated with various concentrations (1, 2, 5 μg protein/mL) of fibrinogen and fibronectin (in sterile water), at 4 °C for 24 h, as described earlier [19]. After that, bacterial suspensions (10^6^ CFU/mL) and compound **2** (30 μM) were added and biofilms were formed for 18 h. Anti-biofilm activity of **2** was measured with the resazurin assay (4.4).

### 3.8. Statistical Analysis and Data Processing

At least four replicates per treatment were included in each plate, and two separate experiments (biological replicates) were always run. Anti-biofilm activities were expressed as inhibition percentages of the untreated biofilms [Equation (1)]. Potencies of the anti-biofilm effects (IC_50_) were calculated from at least 14 concentration points via a non-linear regression analysis (sigmoidal dose-response with variable slope) and the result is presented with 95% confidence intervals. In the case of the cytotoxicity studies, half lethal concentrations (LC_50_) were calculated from viability percentages of untreated controls with Equation (2), using also a similar non-linear regression analysis. Below, μ_max_, and μ_min_ represent the means (average) of the signals (fluorescence of the reduced resazurin, or absorbance of the crystal violet stained biofilms) recorded in untreated biofilms and TSB controls, respectively.

(1) $$\text{Inhibition }\%=[(\mu_{\text{max}}-\mu_{\text{treated well}})/(\mu_{\text{max}}-\mu_{\text{min}})]\times 100\%$$

(2) $$\text{Viability }\%=(\mu_{\text{treated well}}/\mu_{\text{max}})\times 100\%$$

The Biocompatibility Index of compound **2** was calculated according to [28], as the ratio of the LC_50_ value obtained for each tested cell line and the concentration producing 3-log reduction on the viable colonies of biofilms (post-exposure paradigm). In both trials, a similar 24-h exposure to compound **2** was used, to record a more meaningful BI value. A paired t test (two-tailed) was used for the paired comparison in 4.7.5. *p* < 0.05 was regarded as statistically significant. All data processing and statistical analysis was done with Microsoft Excel 2010 software and GraphPad Prism version 5.0 for Mac, GraphPad Software (San Diego, CA, USA), respectively.

## 4. Conclusions

The three abietane-type diterpenes presented herein constitute readily accessible anti-biofilm compounds (in terms of availability and chemical stability). In particular, compound **2** can be obtained from various commercial sources. In contrast, other abietanes such as salvipisone and 4-*epi*-pimaric are not commercially available, and limited amounts have been isolated from plant sources. Moreover, the anti-biofilm potency of compound **2** refers not only to its ability to prevent bacterial colonization, but also to the inhibition of existing biofilms, specifically by acting during the biofilm accumulation stage. A predictive assessment of the therapeutic usefulness made via the calculation of the Biocompatibility Index, showed that compound **2** combines high anti-biofilm efficacy with low cytotoxicity (BI > 1) except for one of the three tested cell lines for which the BI value was not favorable. (+)-Dehydroabietic acid therefore holds great promise as a molecular probe for *in vitro* biofilms studies, and can be used as a potent positive control. Keeping in mind all the elements discussed above, this compound could also serve as well as an inspirational model for the development of potent anti-biofilm drug candidates, but cytotoxicity studies and calculations of the BI values of new chemical derivatives, should be performed early on. Disturbance of flow-grown biofilms by **2** has been further recorded using Bioflux technology (Supplementary Video). Ongoing studies in our laboratory are presently focused on deconvoluting the possible targets of **2**, not only to clarify its mechanism of action, but with the aim of expanding our drug discovery strategies towards target-based approaches as well.

## Figures and Tables

**Table 1:** Chemical structures of the abietane-type diterpenes studied in this contribution.

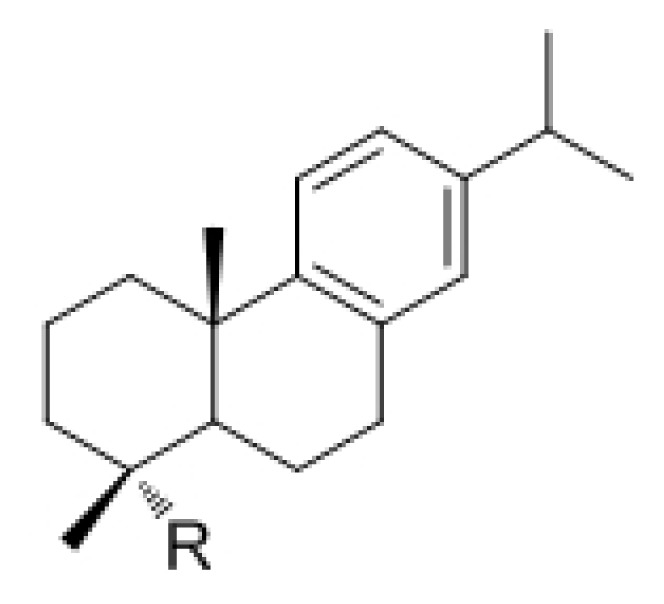			

Code	R	Code	R
**1**	NH_2_	**6**	CH_2_NHCONH-*p*-(C_6_H_4_)OPh
**2**	CO_2_H	**7**	CH_2_NHCONH(C_6_H_3_)(3-OPh)(4-Cl)
**3**	CH_2_NH_2_	**8**	CH_2_NHCONH(C_6_H_2_)(3,6-OMe)(4-Cl)
**4**	CH_2_NHCONH-*p*-(C_6_H_4_)OBn	**9**	CH_2_NHCONH(*tert*-Bu)
**5**	CH_2_NHCONH-*m*-(C_6_H_4_)Cl	**10**	CH_2_NHCONH-5-(benzo-1,3-dioxolyl)

Abbreviations: Bn, benzyl; Ph, phenyl; Bu, butyl; Me, methyl.

**Table 2:** Potencies (IC_50_ values, expressed in μM and mg/L) for compounds **1**–**3** measured for anti-biofilm as well as antibacterial effects on planktonic *S. aureus*. Potency curves were obtained using at least 14 concentration points, with four replicates *per* concentration in two separate experiments (biological replicates) as described in 4.8.

	**Effects on biofilms**				**Effect on planktonic Bacteria**^3^
	IC_50_, μM [mg/L] (95% confidence intervals)				MIC, μM [mg/L]
Cpd	*Prior to* biofilm formation 1		*Post-*biofilm formation 2		Turbidity
	Viability	Biomass	Viability	Biomass	
**1**	124.0 [33.7] (114.6–134.1)	115.6 [31.4] (105.7–126.5)	288.4 [78.3] (266.2–312.5)	293.7 [79.7] (183.1–471.0)	160 [43.4]
**2**	27.8 [8.35] (20.2–38.2)	33.9 [10.2] (23.9–48.3)	112.8 [33.9] (92.9–132.5)	81.2 [24.4] (50.9–129.2)	70 [21.0]
**3**	28.2 [8.05] (26.3–30.2)	17.4 [33.7] (15.5–19.5)	83.4 [23.8] (73.6–94.5)	74.4 [21.2] (70.8–78.2)	40 [11.4]
**Penicillin**	0.13 [0.048] (0.12–0.13)	0.024 [0.0089] (0.02–0.03)	45.2% a	56.5% a	0.12 [0.045]

a Percentual inhibition at 5 mM. Penicillin fails to cause more than 57% of biofilm inhibition in the post-exposure assay, as previously shown by our group in [8];

1 Bacterial suspensions and compounds were added, and biofilms were formed for 18 h (37 °C, 200 rpm);

2 Biofilms were formed for 18 h (37 °C, 200 rpm), compounds were then added for 24 h (37 °C, 200 rpm);

3 Bacterial suspensions and compounds were incubated for 18 h (37 °C, 200 rpm) prior to MIC measurement.

**Table 3:** Biocompatibility index (BI) for compound **2**. Three different values were calculated based on the cytotoxicity results registered in three different cell lines (LC_50_), and the concentration of compound **2** causing a 3-log reduction in the post-exposure anti-biofilm assay (140 μM, corresponding to 42.1 mg/L).

Cell line	Cytotoxicity (LC_50_) μM [mg/L]	Biocompatibility Index *
GT1-7	176.3 [52.9]	1.3
HepG2	153.0 [45.9]	1.1
HL	106.4 [31.9]	0.76

* For the BI calculations, values in mg/L were used.

## Appendix

### Chemistry

All starting synthetic reagents were acquired from Wako Pure Chemical Industries (Osaka, Japan), Fluka (Buchs, Switzerland), and Sigma-Aldrich (St. Louis, MO, USA). Toluene and diethyl ether were distilled from sodium/benzophenone ketyl. All reactions in anhydrous solvents were performed in flame-dried glassware under an inert atmosphere of dry argon. The progress of chemical reactions was monitored by thin-layer chromatography on silica gel 60-F254 plates (Merck, Darmstadt, Germany) using phosphomolybdic acid stain (10% by weight in EtOH) or ninhydrin stain (1.5% by weight in EtOH). Flash SiO_2_ column chromatography was performed with a Merck silica gel 60 (230–400 mesh) or with a Biotage High-Performance Flash Chromatography Sp4-system (Uppsala, Sweden) using a 0.1-mm pathlength flow cell UV-detector/recorder module (fixed wavelength: 254 nm), 12-mm or 25-mm flash cartridges. Purity of all tested compounds was higher than 95% (GC-MS). ^1^H-NMR and ^13^C-NMR spectra were recorded on a Varian Mercury 300 MHz spectrometer (Varian, Palo Alto, CA, USA) as solutions in deuterochloroform, dimethyl sulfoxide-*d*_6_, and acetone-*d*_6_ (Sigma-Aldrich, Schnelldorf, Germany). GC-MS system consisted of a 5890A gas chromatograph and 5970 mass selective detector (Hewlett-Packard, Palo Alto, CA, USA); GC-MS spectra were analyzed with HP Chemstation program. Melting points were measured with an Electrothermal IA 9100 apparatus and are uncorrected.

### Nordehydroabietylamine (**1**)

For preparing nordehydroabietylamine (compound **1**), a mixture of compound **2** (0.304 g, 1.00 mmol), diphenylphosphoryl azide (0.275 g, 1.00 mmol, 1 equiv) and triethylamine (0.101 g, 1.00 mmol, 1 equiv) in anhydrous toluene (10 mL) was stirred, at reflux, for 24 h. Benzyl alcohol (0.541 g, 5.00 mmol, 5 equiv) was added to the reaction mixture and refluxing was continued for 24 h. GC-MS analysis showed the molecular ion [M^+^] of the formed isocyanate (*m/z* 297) while LC-MS analysis showed that of the formed carbamate (*m/z* 405). The burgundy mixture was concentrated under reduced pressure and the resulting crude product was used. After removal of air from the flask by suction, the crude carbamate (0.406 g, 1.00 mmol) was hydrogenated (H_2_, 3 atm) at room temperature in the presence of Pd/C (10%, 0.04 g), in methanol (10 mL), for 16 h. The catalyst was then removed by filtration through a pad of Celite 545 and washed with methanol. The filtrate was concentrated under reduced pressure. The resulting mixture was extracted with dichloromethane (3 × 10 mL). The combined organic layers were dried with anhydrous Na_2_SO_4_, filtrated and evaporated to dryness to give a pale yellow crude solid. The crude product was purified by flash column chromatography (Biotage MPLC, CHCl_3_/MeOH, (10:1) to give a white solid (0.23 g, 85%). *R*_f_ 0.02 (*n*-hexane/EtOAc 2:1); ^1^H NMR (300 MHz, CDCl_3_) δ 8.31 (s, 2H), 6.98 (d, *J* = 8.3 Hz, 1H), 6.89 (d, *J* = 8.3 Hz, 1H), 6.82 (s, 1H), 2.94–2.58 (m, 2H), 2.13 (d, *J* = 12.4 Hz, 1H), 1.99 (dd, *J* = 49.8, 12.1 Hz, 3H), 1.51 (s, 4H), 1.28 (s, 4H), 1.25 (s, 3H), 1.21 (s, 2H), 1.07 (s, 3H), 0.98–0.47 (m, 2H); ^13^C NMR (75 MHz, CDCl_3_) δ 152.80, 145.89, 134.78, 127.13, 124.73, 124.00, 57.39, 48.07, 38.07, 37.24, 33.74, 31.79, 29.88, 25.07, 22.86, 19.62, 19.16, 18.36, 14.32; GC-MS *m/z* 271 [M^+^] (*t*_R_ = 12.6 min).

### (+)-Dehydroabietic acid (**2**)

(+)-Dehydroabietic acid (compound **2**) was commercially purchased (Wako Pure Chemical Industries, Ltd, 85%–90%) and recrystallized from *n*-hexane and ethyl acetate to give white crystals. *R*_f_ 0.62 (*n*-hexane/EtOAc 2:1); mp 168–171 °C; ^1^H NMR (300 MHz, CDCl_3_) δ 11.64 (s, 1H), 7.17 (d, *J* = 8.2 Hz, 1H), 7.01 (d, *J* = 8.2 Hz, 1H), 6.90 (s, 1H), 3.01–2.78 (m, 2H), 2.32 (d, *J* = 12.7 Hz, 1H), 2.26 (dd, *J* = 12.4, 2.1 Hz, 1H), 1.96–1.56 (m, 6H), 1.56 (m, 3H), 1.30 (s, 3H), 1.25 (s, 3H), 1.23 (s, 3H), 1.22 (s, 3H); ^13^C NMR (75 MHz, CDCl_3_) δ 185.36, 146.95, 145.93, 134.89, 127.10, 124.30, 124.08, 47.63, 44.80, 38.12, 37.07, 36.95, 33.67, 30.20, 25.33, 24.18, 21.97, 18.74, 16.41; GC-MS *m/z* 300 [M^+^] (*t*_R_ = 8.17 min); IR (KBr, cm^−1^): 3100 br m, 2958 br m, 2931, 2869, 1694 C=O, 1459, 1280, 1190, 949, 819.

### (+)-Dehydroabietylamine (**3**)

(+)-Dehydroabietylamine (compound **3**) was purified by a reported crystallization method [33] with slight modifications. The crude (+)-dehydroabietylamine (Sigma-Aldrich, St. Louis, MO, USA) was estimated to have a purity of approx. 60% by GC-MS. A solution of glacial acetic acid (7.5 mL) in toluene (75 mL) was added dropwise over a period of 40 min to a cooled (0 °C) solution of crude compound (35.0 g, 0.13 mol) in toluene (200 mL). The mixture was stirred at 10 °C for 2 h, and the resulting precipitate was collected under reduced pressure, washed with cold toluene (75 mL) and dried. The white solid was recrystallized from toluene (150 mL) to yield dehydroabietylammonium acetate (37.5 g, 70%) as fine, white needles, mp 141–143 °C, [α]^D^_21_ + 31 (*c* 2.5, MeOH, mp 141–143.5 °C, [α]^D^_25_ + 30.5) [33]. Dehydroabietylammonium acetate (25.0 g, 0.145 mmol) was dissolved in warm water (150 mL) and cooled to room temperature before adding 1 M sodium hydroxide (20 mL). The resulting solution was stirred for 1 h before extracting the free amine into diethyl ether (250 mL). The organic solution was dried with anhydrous sodium sulphate, filtered and evaporated to dryness Residual solvent was removed *in vacuo* to yield (+)-dehydroabietylamine (39.7 g, 96%) as a yellow oil, which was washed with diethyl ether to give a white solid. *R*_f_ 0.13 (*n*-hexane/EtOAc 2:1); mp 41–43 °C, [33], mp 41 °C); ^1^H NMR (300 MHz, CDCl_3_) δ 7.19 (d, *J* = 8.2 Hz, 1H), 7.00 (dd, *J* = 8.2, 2.0 Hz, 1H), 6.90 (s, 1H), 2.98–2.77 (m, 3H), 2.61 (d, *J* = 13.3 Hz, 1H), 2.40 (d, *J* = 13.3 Hz, 1H), 2.29 (dt, *J* = 12.0, 3.1 Hz, 1H), 1.87–1.61 (m, 5H), 1.52 (dd, *J* = 10.6, 4.1 Hz, 1H), 1.43 (d, *J* = 4.1 Hz, 1H), 1.34 (td, 14.7, 4.1 Hz, 3H), 1.24 (br. s, 3H), 1.23 br. s, 3H), 0.89 br. s, 3H); ^13^C NMR (75 MHz, CDCl_3_) δ 147.66, 145.70, 134.88, 126.98, 124.43, 124.00, 54.03, 45.04, 38.73, 37.57, 37.39, 35.39, 33.62, 30.36, 25.44, 24.19, 24.17, 18.95, 18.82; IR (KBr, cm^−1^): 3393.5 (N–H), 2956, 2923, 2864, 1611, 1560, 1497, 1457; GC-MS *m/z* 285 [M^+^] (*t*_R_ = 6.81 min); LC-MS *m/z* 286.3 [M + H]^+^ (*t**_R_* = 15.4 min, 98.7%).

Compounds **4**–**10** were synthesized from compound **3** via the isocyanate method [36], with isocyanate (1.1 eq), in tetrahydrofuran, at r.t. for 24 h.

### 1-[4-(Benzyloxy)phenyl]-3-[[(1*R*,4a*S*)-7-isopropyl-1,4a-dimethyl-1,2,3,4,4a,9,10,10aoctahydrophenanthren- 1-yl]methyl]urea (**4**)

*R*_f_ 0.53 (*n*-hexane/EtOAc, 2:1); mp 141–143 °C; ^1^H NMR (300 MHz, DMSO-*d*_6_) *δ* 8.16 (s, 1H), 7.55–6.50 (m, 10H), 6.01 (s, 1H), 5.01 (s, 2H), 3.32 (d, *J* = 1.6 Hz, 2H), 3.13 (dd, *J* = 13.4, 5.8 Hz, 1H), 2.96–2.61 (m, 3H), 2.26 (d, *J* = 12.4 Hz, 1H), 2.02–0.50 (m, 18H); ^13^C NMR (75 MHz, DMSO-*d*_6_) δ 155.43, 152.75, 147.09, 144.93, 137.36, 134.50, 133.99, 128.35, 127.68, 127.59, 126.40, 124.10, 123.55, 118.87, 114.95, 69.38, 49.06, 43.96, 38.10, 37.12, 36.94, 35.39, 32.88, 29.73, 25.13, 23.94, 18.85, 18.26; ^13^C NMR DEPT (75 MHz, DMSO-*d*_6_) δ 128.36, 127.69, 127.60, 126.41, 124.11, 123.56, 118.88, 114.96, 69.38, 49.06, 43.96, 38.10, 32.88, 29.73, 25.13, 23.95, 18.86, 18.27; IR (KBr, cm^−1^): 3343 br m N–H, 2925 br m, 1645 C=O, 1564, 1511, 1223, 826; LC-MS (508.31) *m*/*z* 509.3 [M + H]^+^ (*t**_R_* = 22.1 min, 96.2%).

### 1-(3-Chlorophenyl)-3-[[(1*R*,4a*S*)-7-isopropyl-1,4a-dimethyl-1,2,3,4,4a,9,10,10aoctahydrophenanthren- 1-yl]methyl]urea (**5**)

*R*_f_ 0.40 (*n*-hexane/EtOAc, 2:1); mp 102–104 °C; ^1^H NMR (300 MHz, Acetone-*d*_6_) δ 8.05 (s, 1H), 7.79–7.66 (m, 1H), 7.25–7.09 (m, 3H), 6.99–6.92 (m, 1H), 6.89 (m, 1H), 6.86 (s, 1H), 5.89 (s, 1H), 3.36–3.25 (m, 1H), 3.00 (dd, *J* = 13.8, 6.4 Hz, 1H), 2.87–2.68 (m, 3H), 2.28 (dt, *J* = 15.6, 7.9 Hz, 1H), 2.00–1.90 (m, 1H), 1.87–1.54 (m, 3H), 1.52–1.25 (m, 3H), 1.20 (s, 6H), 1.17 (s, 3H), 0.94 (s, 3H); ^13^C NMR (75 MHz, Acetone-*d*_6_) δ 156.03, 148.17, 146.18, 143.13, 135.60, 134.71, 130.77, 127.55, 124.99, 124.49, 121.79, 118.45, 116.90, 50.46, 45.44, 39.31, 38.31, 38.12, 36.67, 34.26, 30.90, 25.67, 24.38, 19.58, 19.36; IR (KBr, cm^−1^): LC-MS (438.24) *m*/*z* 439.3 [M + H]^+^, 461.3 [M + Na]^+^ (*t**_R_* = 22.4, min, 95.8%).

### 1-[[(1*R*,4a*S*)-7-isopropyl-1,4a-dimethyl-1,2,3,4,4a,9,10,10a-octahydrophenanthren-1-yl]methyl]-3-(4- phenoxyphenyl)urea (**6**)

*R*_f_ 0.64 (*n*-hexane/EtOAc, 2:1); mp 162–164 °C; ^1^H NMR (300 MHz, DMSO-*d*_6_) δ 8.36 (s, 1H), 7.32 (m, 4H), 7.13 (d, *J* = 8.2 Hz, 1H), 7.09–7.00 (m, 1H), 6.90 (t, *J* = 9.4 Hz, 5H), 6.82 (s, 1H), 6.08 (t, *J* = 6.0 Hz, 1H), 3.27–3.05 (m, 1H), 2.77 (m, 5H), 2.25 (d, *J* = 12.6 Hz, 1H), 1.72 (m, 7H), 1.13 (s, 6H), 1.11 (s, 3H), 0.85 (s, 3H); ^13^C NMR (75 MHz, DMSO-*d*_6_) δ 157.87, 155.32, 149.68, 147.08, 144.94, 136.75, 134.49, 129.82, 126.41, 124.09, 123.56, 122.51, 119.88, 118.89, 117.31, 49.04, 43.97, 38.10, 37.14, 36.94, 35.38, 32.88, 29.73, 25.12, 23.95, 18.84, 18.31; IR (KBr, cm^−1^): 3334, 2957, 2924.0, 2866, 1645, 1562, 1488, 1226, 1089, 832; LC-MS (496.31) *m*/*z* 497.3 [M + H]^+^ (*t**_R_* = 22.3 min, 96.8%).

### 1-(4-Chloro-2-phenoxyphenyl)-3-[[(1*R*,4a*S*)-7-isopropyl-1,4a-dimethyl-1,2,3,4,4a,9,10,10aoctahydrophenanthren- 1-yl]methyl]urea (**7**)

*R*_f_ 0.88 (*n*-hexane/EtOAc, 2:1); mp 240–243 °C; ^1^H NMR (300 MHz, DMSO-*d*_6_) δ 8.37 (s, 1H), 8.34 (d, *J* = 2.5 Hz, 1H), 7.34 (t, *J* = 8.0 Hz, 2H), 7.13 (d, *J* = 8.1 Hz, 2H), 7.07–6.91 (m, 3H), 6.87 (dd, *J* = 8.6, 2.6 Hz, 2H), 6.83 (s, 1H), 6.72 (d, *J* = 8.6 Hz, 1H), 4.01 (s, 1H), 3.19 (d, *J* = 13.7 Hz, 1H), 2.78 (m, 4H), 2.25 (d, *J* = 12.2 Hz, 1H), 1.72 (d, *J* = 71.2 Hz, 5H), 1.31 (m, 4H), 1.14 (s, 6H), 1.13 (br. s, 3H), 0.86 (s, 3H); ^13^C NMR (75 MHz, DMSO-*d*_6_) δ 156.24, 154.94, 147.00, 144.92, 143.61, 134.41, 133.26, 130.01, 127.39, 126.38, 124.05, 123.84, 123.53, 120.47, 118.70, 118.30, 49.27, 44.12, 38.00, 37.06, 36.94, 35.46, 32.84, 29.72, 25.09, 23.89, 18.72, 18.33, 18.17; IR (KBr, cm^−1^): 3346 br m, N–H, 2958, 2934, 2888, 1673 C=O, 1591, 1556, 1522, 1183, 860, 749, 687; LC-MS (530.27) *m*/*z* 531.2 [M + H]^+^ (*t**_R_* = 22.3 min).

### 1-(4-Chloro-2,5-dimethoxyphenyl)-3-[[(1*R*,4a*S*)-7-isopropyl-1,4a-dimethyl-1,2,3,4,4a,9,10,10aoctahydrophenanthren- 1-yl]methyl]urea (**8**)

*R*_f_ 0.29 (*n*-hexane/EtOAc, 2:1); mp 223–225 °C; ^1^H NMR (300 MHz, Acetone-*d*_6_) δ 8.36 (s, 1H), 7.49 (s, 1H), 7.16 (d, *J* = 8.2 Hz, 1H), 6.95 (d, *J* = 8.2 Hz, 1H), 6.87 (s, 1H), 6.77 (d, *J* = 9.5 Hz, 1H), 6.21 (t, *J* = 6.5 Hz, 1H), 3.83 (s, 3H), 3.81 (s, 3H), 3.34 (dd, *J* = 13.8, 6.4 Hz, 1H), 2.97 (dd, *J* = 13.7, 6.4 Hz, 1H), 2.79 (m, 5H), 2.31 (d, *J* = 12.9 Hz, 1H), 12.00–1.23 (m, 9H), 1.20 (s, 3H), 1.19 (s, 3H), 1.17 (s, 3H), 0.95 (s, 3H); ^13^C NMR (75 MHz, Acetone-*d*_6_) δ 156.10, 150.32, 148.30, 148.10, 146.21, 135.72, 127.59, 125.00, 124.48, 120.30, 117.67, 116.16, 113.55, 98.56, 56.99, 56.55, 50.47, 45.20, 39.36, 38.34, 38.13, 36.68, 34.29, 30.83, 25.66, 24.37, 19.58, 19.41; IR (ATR, cm^−1^): 3346 br m, N–H, 2967, 2935, 2868, 1739 C=O, 1643, 1501, 1389, 1203, 1034, 851, 767, 688; LC-MS (498.26) *m*/*z* 499.3 [M + H]^+^, 521.3 [M + Na]^+^ (*t**_R_* = 20.9 min)

### 1-(*tert*-Butyl)-3-[[(1*R*,4a*S*)-7-isopropyl-1,4a-dimethyl-1,2,3,4,4a,9,10,10a-octahydrophenanthren-1- yl]methyl]urea (**9**)

*R*_f_ 0.55 (*n*-hexane/EtOAc, 2:1); mp 181–183 °C; ^1^H NMR (300 MHz, DMSO-*d*_6_) δ 7.14 (dd, *J* = 8.2, 3.3 Hz, 1H), 6.94 (d, *J* = 8.1 Hz, 1H), 6.83 (s, 1H), 5.60 (s, 2H), 3.33 (d, *J* = 1.5 Hz, 1H), 3.01 (d, *J* = 13.5 Hz, 1H), 2.73 (m, 3H), 2.25 (d, *J* = 12.2 Hz, 1H), 2.05–1.19 (m, 9H), 1.18 (s, 9H), 1.13 (s, 6H), 0.81 (s, 3H). ^13^C NMR (75 MHz, DMSO-*d*_6_) δ 157.39, 147.15, 144.88, 134.60, 126.37, 124.12, 123.48, 49.00, 48.84, 43.92, 38.14, 37.00, 36.92, 35.44, 32.87, 29.73, 29.28, 25.14, 23.98, 23.93, 18.87, 18.31, 18.24; IR (KBr, cm^−1^): 3332 br m, N–H, 2961, 2923, 2867, 1632 C=O, 1568, 1451, 1384, 1244, 1157, 819, 674; LC-MS (498.26) *m*/*z* 499.3 [M + H]^+^ (*t**_R_* = 20.9 min, 99.0%).

### 1-(Benzo[*d*][1,3]dioxol-5-yl)-3-[[(1*R*,4a*S*)-7-isopropyl-1,4a-dimethyl-1,2,3,4,4a,9,10,10aoctahydrophenanthren- 1-yl]methyl]urea (**10**)

*R*_f_ 0.36 (*n*-hexane/EtOAc, 2:1); mp 157–159 °C; ^1^H NMR (300 MHz, CDCl_3_) δ 7.31–7.22 (m, 1H), 7.20–7.10 (m, 1H), 7.03–6.93 (m, 1H), 6.86 (dd, *J* = 5.6, 1.8 Hz, 1H), 6.67 (d, *J* = 8.2 Hz, 1H), 6.60 (dd, *J* = 8.2, 2.0 Hz, 1H), 6.41–6.27 (m, 1H), 5.93–5.82 (m, 1H), 4.83 (t, *J* = 6.2 Hz, 1H), 3.20–3.02 (m, 2H), 2.93–2.68 (m, 2H), 2.27 (d, *J* = 12.7 Hz, 1H), 1.93–1.53 (m, 4H), 1.38 (ddd, *J* = 15.5, 11.0, 4.9 Hz, 2H), 1.26–0.76 (m, 11H); ^13^C NMR (75 MHz, CDCl_3_) δ 156.71, 148.32, 147.36, 145.76, 144.91, 134.96, 132.45, 127.06, 124.39, 123.99, 116.09, 108.48, 105.24, 101.53, 50.90, 45.59, 38.65, 37.74, 37.64, 36.35, 33.62, 30.45, 25.45, 24.18, 19.11, 18.83, 18.77; IR (KBr, cm^−1^): 3380 br m, N–H, 2950, 2914, 2872, 1650 C=O, 1573, 1503, 1490, 1384, 1251, 1210, 1038, 832, 800, 667; HRMS-ESI (*m*/*z*): [M + H]^+^ calcd. 449.2804; found 449.2809.

### Supplementary Video

Movie showing the interaction of compound **2** with *S. aureus* biofilms grown under flow conditions using Bioflux 200 (Fluxion Biosciences, CA, USA). Biofilms were grown in 48-well microplates (20 dyne/cm^2^) having microfluidic channels (370 μm × 70 μm) that were inoculated with *S. aureus* (10^6^ CFU/mL) in 1:10 diluted TSB. Inoculation was performed by pumping at 2 dyne/cm^2^ for 2 s, from the “outlet” wells to the “inlet” wells to prevent contamination of the inlet wells. Following 1 h incubation for biofilm attachment (on static conditions) at 37 °C, flow was resumed at 0.4 dyne/cm^2^ for 18 h from inlet to outlet wells. After biofilm formation, compound **2** (100 μM, in 1:10 TSB) was added into the inlet wells and flow was restarted (0.2 dyne/cm^2^) for additional 24 h. The video (13 s) was automatically created from timelapse images taken every 1 s with a coupled Transmitted Light Evos ×l microscope (AMG, WA, USA) with 40× objective, 15 min after starting the exposure to **2**.
